# Editorial: Mesenchymal stem cell-derived extracellular vesicles: considerations and therapeutic applications

**DOI:** 10.3389/fcell.2024.1377197

**Published:** 2024-02-23

**Authors:** Faezeh Shekari, Anna Meyfour, Owen G. Davies, Émilie Velot

**Affiliations:** ^1^ Department of Stem Cells and Developmental Biology, Cell Science Research Center, Royan Institute for Stem Cell Biology and Technology, Academic Center for Education, Culture and Research (ACECR), Tehran, Iran; ^2^ Advanced Therapy Medicinal Product Technology Development Center (ATMP-TDC), Cell Science Research Center, Royan Institute for Stem Cell Biology and Technology, Academic Center for Education, Culture and Research (ACECR), Tehran, Iran; ^3^ Basic and Molecular Epidemiology of Gastrointestinal Disorders Research Center, Research Institute for Gastroenterology and Liver Diseases, Shahid Beheshti University of Medical Sciences, Tehran, Iran; ^4^ School of Sport, Exercise and Health Sciences, Loughborough University, Loughborough, United Kingdom; ^5^ Université de Lorraine, French National Centre for Scientific Research (CNRS), Laboratory of Molecular Engineering and Physiopathology (IMoPA), Nancy, France

**Keywords:** extracellular vesicle (EV), exosomes, therapeutic, mesenchymal stromal (or stem) cells, cell-free therapy

Mesenchymal stem cells (MSCs) have emerged as pivotal players in cell therapy studies, orchestrating an array of paracrine effects primarily through the dynamic release of extracellular vesicles (EVs). These EVs can be sourced from diverse entities such as plasma membrane (ectosomes), multivesicular bodies (exosomes), or apoptotic cells (apoptotic bodies). Each encapsulates a rich spectrum of constituents that includes proteins, nucleic acids, lipids, and metabolites. Released under both physiological and non-physiological conditions, EVs serve as essential mediators in cellular communication. Over the past decade, MSC-derived EVs (MSC-EVs) have demonstrated considerable promise, with positive pro-therapeutic effects observed across an extensive array of diseases, including autoimmune, brain, cancer, eye, gastrointestinal, heart, liver, musculoskeletal, pancreas, nervous system, respiratory system, reproductive system, skin, urinary system, and vascular-related conditions. The objective of this Research Topic is to compile novel research publications that broadly explore the potential therapeutic applications of MSC-EVs, provide insightful considerations for their use as drug delivery vehicles, and address current challenges associated with large-scale production.

This Research Topic reports on multiple MSC sources being applied for MSC-EVs production, encompassing bone marrow (Cavallero et al.; Dedier et al.), gingiva (Dedier et al.), periodontal ligament (Lan et al.), induced pluripotent stem cells (Tertel et al.), adipose tissue (Lim et al.), and Wharton’s jelly (Drobiova et al.). This diversity highlights the range of MSC-EV sources currently being explored therapeutically.

The studies presented in this Research Topic demonstrate that MSC-EVs exhibit considerable therapeutic potential, with reported angiogenic, osteogenic and anti-inflammatory properties. Research by Dedier et al. explored the pro-healing capabilities of MSC secretomes from gingiva and bone marrow stem cells, upon interleukin (IL)-1β pro-inflammatory priming. Results underscore the anti-inflammatory effects of MSCs and their potential to enhance the current treatment of severe wounds (Dedier et al.). Notably, IL-6 enrichment in the secretome of IL-1β-primed MSCs was implicated in the anti-inflammatory effects observed (Dedier et al.). Cavallero et al. described enhancement in the pro-angiogenic potential of EVs through MSC preconditioning with Tumor Necrosis Factor alpha (TNF)α and Interferon (IFN)γ. Lan et al. presented a study in which EVs were isolated from curcumin-pretreated periodontal ligament stem cells to examine their impact on proliferation, migration, and osteogenic capacity, and suggests the potential of these EVs in promoting osteogenic differentiation, offering valuable insights for periodontal disease treatment.

To manufacture MSC-EVs (Tertel et al.), discussed protocols for deriving induced mesenchymal stem cells (iMSCs) from good manufacturing practice (GMP)-compliant induced pluripotent stem cells (iPSCs). Outcomes highlighted functional variability in the resulting iMSC-EV products that mirrored those of bone marrow MSC counterparts. Their findings emphasize a critical need for batch-to-batch functional testing to identify effective MSC-EV products under international GMP standards.

Three review articles presented in this Research Topic provide an in-depth exploration of EV isolation methodologies, and the therapeutic potential of MSCs and their EVs in addressing complex conditions such as neural pathologies and Acute Respiratory Distress Syndrome (ARDS). In a comprehensive review (Lim et al.), delved into the intricacies of EV isolation methodologies, explored distinctions related to adipose tissue-MSC, and discussed the therapeutic potential of EVs in treating neural pathologies. The review further provides insight into the scalability and standardization of small EV production for clinical applications. A review by Drobiova et al. centers on recent advances in research on the secretome of Wharton’s jelly mesenchymal stem cells. The discussion provides a comparative analysis of WJ-MSCs’ secretome components with those of other MSCs, shedding light on therapeutic applications across various diseases. Zhuang et al. contributed to a comprehensive overview of the therapeutic potential of MSCs and their EVs in treating ARDS, summarizing research progress over the past 5 years. They highlighted the protective role of MSCs and their EVs in the pathophysiology of ARDS, in terms of antimicrobial activity, restoration of alveolar-capillary barrier integrity, immunomodulatory, inhibition of cell death, and regulation of autophagy.

In summary, this special edition emphasizes the considerable impact of MSC-EVs on the field of cell-free therapy, highlighting their varied paracrine effects and therapeutic promise across a broad range of medical conditions. It makes a substantial contribution to advancing our understanding of MSC-EVs, providing valuable insights into their therapeutic potentials and tackling the obstacles linked to large-scale production.

